# Biofabrication of Anisotropic Gold Nanotriangles Using Extract of Endophytic *Aspergillus clavatus* as a Dual Functional Reductant and Stabilizer

**DOI:** 10.1007/s11671-010-9743-6

**Published:** 2010-08-28

**Authors:** Vijay C Verma, Santosh K Singh, Ravindra Solanki, Satya Prakash

**Affiliations:** 1 Centre of Experimental Medicine and Surgery, Institute of Medical Sciences, Banaras Hindu University, Varanasi 221005, India; 2 School of Material Science and Technology, Institute of Technology, Banaras Hindu University, Varanasi 221005, India; 3 National Facility for Tribal and Herbal Medicine, Institute of Medical Sciences, Banaras Hindu University, Varanasi 221005, India

**Keywords:** *Azadirachta indica*, Gold nanotriangles, Endophytic fungi, XRD, AFM, *Aspergillus clavatus*

## Abstract

Biosynthesis of metal and semiconductor nanoparticles using microorganisms has emerged as a more eco-friendly, simpler and reproducible alternative to the chemical synthesis, allowing the generation of rare forms such as nanotriangles and prisms. Here, we report the endophytic fungus *Aspergillus clavatus*, isolated from surface sterilized stem tissues of *Azadirachta indica* A. Juss., when incubated with an aqueous solution of chloroaurate ions produces a diverse mixture of intracellular gold nanoparticles (AuNPs), especially nanotriangles (GNT) in the size range from 20 to 35 nm. These structures (GNT) are of special interest since they possess distinct plasmonic features in the visible and IR regions, which equipped them with unique physical and optical properties exploitable in vital applications such as optics, electronics, catalysis and biomedicine. The reaction process was simple and convenient to handle and was monitored using ultraviolet–visible spectroscopy (UV–vis). The morphology and crystalline nature of the GNTs were determined from transmission electron microscopy (TEM), atomic force spectroscopy (AFM) and X-ray diffraction (XRD) spectroscopy. This proposed mechanistic principal might serve as a set of design rule for the synthesis of anisotropic nanostructures with desired architecture and can be amenable for the large scale commercial production and technical applications.

## Introduction

At present, there is a greater need to develop safe, reliable, clean and eco-friendly methods for the preparation of nanoparticles and other high structured nanomaterials. With the rapid development of new chemical/physical methods, concern for environmental contaminations is regularly heightened as the chemical procedures involved in the synthesis of nanomaterials generates a large amount of hazardous by-products. Thus, there is an urgent need for 'green chemistry' that includes clean, nontoxic and environment-friendly methods of nanoparticle synthesis with precise control over the shape and size. In the recent years, 'green synthesis' of the nanoparticles has paid much more attention in the rapidly growing area of nanoscience and nanotechnology [1-5]. Utilization of cheap nontoxic chemicals, eco-friendly solvents and renewable materials are some of the pivotal issues that merit important concern in a green synthesis strategy for nanomaterials. In this context, biological synthesis of nanoparticles as an emerging highlight of the intersection of nanotechnology and biotechnology has received increasing attention to come up the need of environmentally benign technologies in nanomaterial synthesis, not only because it reduce the use and generation of hazardous substances to human health and environment but also in providing the facile and convenient entry to produce multiple inorganic nanoparticles [6]. Thus, synthesis of nanomaterials using microorganisms is compatible with the green chemistry principles, resulted in a surge of interest in scientists towards biological systems for inspiration [7-9]. Many microbes are known to produce highly structured metallic nanoparticles with very similar properties to that of chemically synthesized materials, while having precise control over size, shape and monodispersity. The magnetosome or the magnetotactic bacteria synthesize the magnetic nanoparticles in nature since long back, which is a very good biosystem to learn the basic principles of biofabrication [10]. Many prokaryotes like *Pseudomonas stutzeri*[11] and *Schizosacchromyces pombe*[12] are reported to produce silver and cadmium nanocrystals within their periplasmic spaces. Besides these, there are several other eukaryotic microbes such as fungi *Verticillium* and *Fusarium* that synthesize the gold nanoparticles with variable shape and size [13,14]. These all examples rectify the importance of bio-systems to get inspiration in fabricating nanomaterials.

In this report, we present the single step 'green synthesis' protocol for biofabricating highly anisotropic, monocrystalline gold nanotriangles utilizing extracts of endophytic (endophytes are microbe that resides within the internal living tissues of higher plants as endosymbionts) fungi *Aspergillus clavatus*, which was isolated from the surface-sterilized stem tissues of *Azadirachta indica* A. Juss. Earlier, there are many other species of *Aspergillus* have been reported of their potential to synthesize silver and gold nanoparticles such as *Aspergillus niger*[15], *A. flavus*[16], *A. Fumigatus*[17], *A. oryzae* var. *Viridis*[9]. Although this endophytic microbe was earlier investigated by our group for the biofabrication of silver nanoparticles [4], but no reports are available about their potential in biofabrication of gold nanoaparticles. This strain is largest among the *Aspergillus* spp. and conidiophores can be seen from unaided eye. This is first ever report of an endophytic *A. Clavatus*, in bio-fabricating gold nanoparticles, although some other endophytic fungi like *Colletotrichum* sp. from *Pelargonium graveolens* leaves are reported for gold bio-fabrication [18]. Most of the earlier works are emphasizing with the size of nanoparticles in contrast to this report which shows a precise control not only over size but also its shape specially nanotriangle.

## Experimental Details

### Isolation of Endophytic *Aspergillus clavatus*

The host plant *Azadirachta indica* A. Juss. was surveyed, and samples were randomly collected from within the campus premises of Banaras Hindu University, Varanasi, India. The stem tissues were collected with cut ends sealed with parafilms™ and collected in paper bags. The samples were than washed properly in running tap water for 5–8 min followed by rinse in sterile distilled water to remove the adhered debris and spores. After successive surface sterilization in 75% ethanol (5 min), the stem tissues were rinsed three times in sterilized distilled water and aseptically cut into small pads (0.5 × 0.5 cm^2^). The small pads were carefully placed onto PDA plates and incubated at 25°C for 20 days until the mycelia of endophytic fungi appeared. Each isolate was then grown and examined to ascertain that it originated from a single spore. Based on literature and other morphotaxonomic features under microscope (Nikon Eclipse E-600), one of the strains is identified as *Aspergillus clavatus*.

### Biological Synthesis of Gold Nanotriangles

The endophytic *Aspergillus clavatus* strain was grown in 500-ml Erlenmeyer flasks containing 200 ml MGYP medium which is composed of malt extract (0.3%), glucose (1%), yeast extract (0.3%) and peptone (0.5%), and after adjusting the pH of the medium to 7.0, the culture was grown with continuous shaking on a rotary shaker (200 rpm) at 27°C for 8 days. After the fermentation of the culture, biomass was harvested by centrifugation (5,000 rpm) at 20°C for 20 min, and then the mycelia were washed thrice with sterile distilled water under aseptic conditions. In the present study, we have used both the biomass (wet mycelia) and the culture-free spent medium (culture extract) as reducing agent. The thoroughly washed and harvested mycelial biomass (10 g wet weight) was suspended in 100 ml of aqueous 1 mM HAuCl_4_ in 500-ml Erlenmeyer's flasks. This reaction mixture was then put onto a shaker at room temperature and 200 rpm. The reaction mixture was routinely monitored by visual colour change as well as periodic sampling of aliquots (2 ml) of the reaction mixture and measuring the UV–vis spectra on a Hitachi dual-beam spectrophotometer (Hitachi, UV-2910) operated at a resolution of 1 nm. Similarly, the broth extract of the endophytic strain is also utilized for bioreduction of aqueous gold ion solution. In a flask, 90 ml of aqueous 1 mM HAuCl_4_ solution was taken and 10 ml of fungal extract solution is added, thereafter the reaction mixture is placed on rotary shaker as in conditions similar to the biomass-based reduction.

### Characterization of Gold Nanotriangles

Once the reactions in the flasks have been completed, the nanoparticles formed were accordingly characterized with TEM, XRD and AFM. For XRD studies, the biomass of fungal mycelia after the reaction has been taken and dried in sterile condition in hot air oven and ground into fine powder. The characterization of gold nanoparticles was carried out by XRD (Cu-Kα radiation source) using a 12-kW rotoflux rotating Cu anode (Rigaku Tokyo, Japan) powder diffractometer (RINT 2000/PC series) operating in Bragg–Brentano geometry and fitted with a curved crystal graphite monochromator in the diffraction beam and a high temperature attachment. For TEM analysis, the samples were prepared by placing 5 μl of gold nanoparticle suspension on a 300-mesh carbon-coated copper grid, and the solution was allowed to stand for 5 min, then excess solution was removed carefully, and the grid was allowed to dry for an additional 5 min; the average size and size distributions of gold nanoparticles were determined by processing the TEM images with image processing software on a Tecnai G-20 transmission electron microscope, a 200-kV TEM with a W-source and an ultra high-resolution pole piece with a point–point resolution of 1.9 A° (TEM, Tecnai [FEI]-12v.G-20). Surface topology was measured by atomic force microscopy (AFM) in the contact mode on a VEECO Digital Instruments multimode scanning probe microscope equipped with a Nanoscope IV controller.

## Results and Discussion

**Figure 1:**
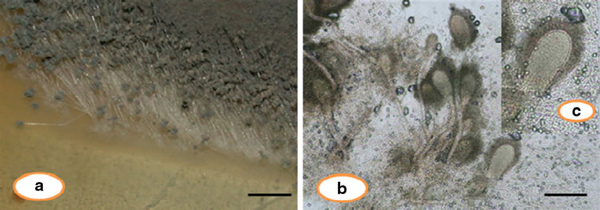
**The endophytic *Aspergillus clavatus*, isolated from surface sterilized stem tissues *Azadirachta indica* A. Juss**. **a** Bunches of conidiophores as visualized onto petriplate, **b** the club-shaped conidiophores, **c** stipes smooth walled and hyaline (*Bar* represents magnifications ×40 for **a** and **b**, while ×100 for **c**).

**Figure 2:**
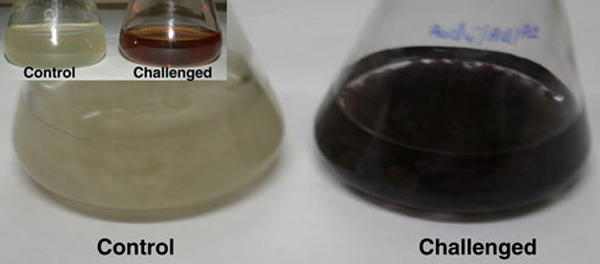
**The flasks containing the biomass of the *Aspergillus clavatus* biomass in sterile distilled water (Control) and in aqueous solution of 1 mM HAuCl_4_ after the reaction of 72 h (the biomass colour changed into *dark purple*)**. Inset shows the pictures of fermentation broth of *Aspergillus clavatus* challenged by 1 mM HAuCl_4_.

**Figure 3:**
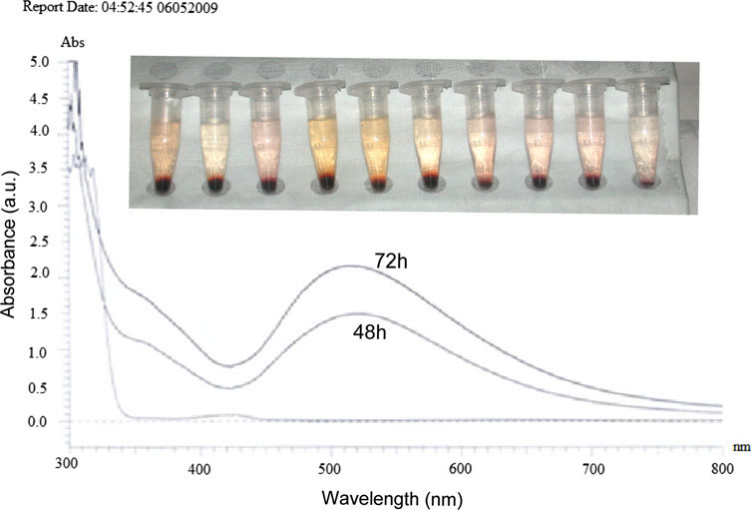
**UV–vis spectra recorded as a function of reaction time of an aqueous solution of 1 mM HAuCl_4_ with the filtrate of the fungal biomass**. The spectra show a sharp absorption λ_max_ 540 at 48 h reaction periods, which intensifying with the 72 h reaction time. The inset shows vials containing the filtrate of *A. clavatus* collected during the reaction period, rapid change in colour pattern due to SPR are clearly visible.

**Figure 4:**
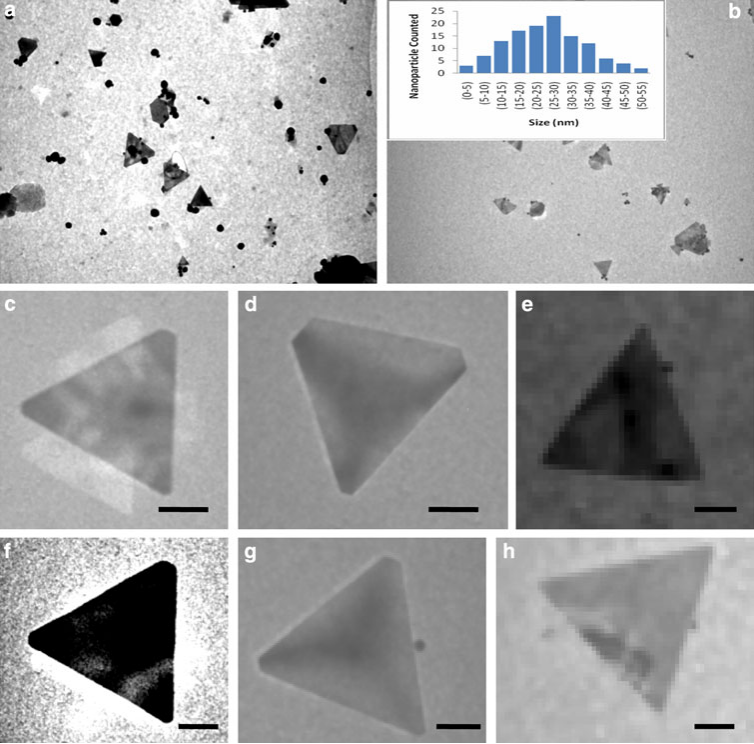
**The TEM images of gold nanotriangles synthesized by the reduction of 1 mM HAuCl_4_ by endophytic *A. clavatus* (a–b)**. The inset in (**b**) shows the histogram analysis for edge-to-edge length of the gold nanotriangles. The different types of edge/tip margins of gold nanotriangles were observed such as sharp-edged triangles (**e**, **h**), truncated triangles (**c**, **f**, **g**) and snipped triangles (**d**), (*bar* represent 100 nm for **a** and **b**, while 50 nm for **c**–**h**).

**Figure 5:**
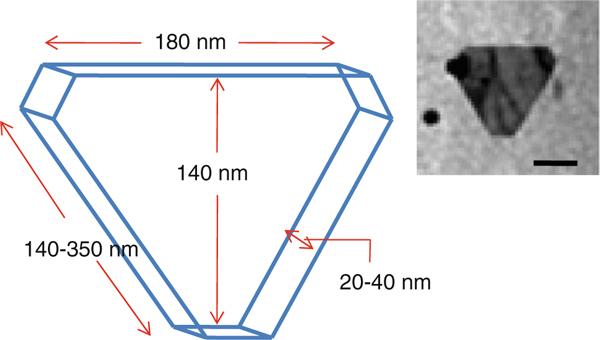
**Measurement of a relatively large, single-snipped gold nanotriangle shows a height of about 140 nm, while the edge length varies from 140–350 nm**. Although several other triangles have much smaller dimensions then this one, which was also observed with AFM study.

**Figure 6:**
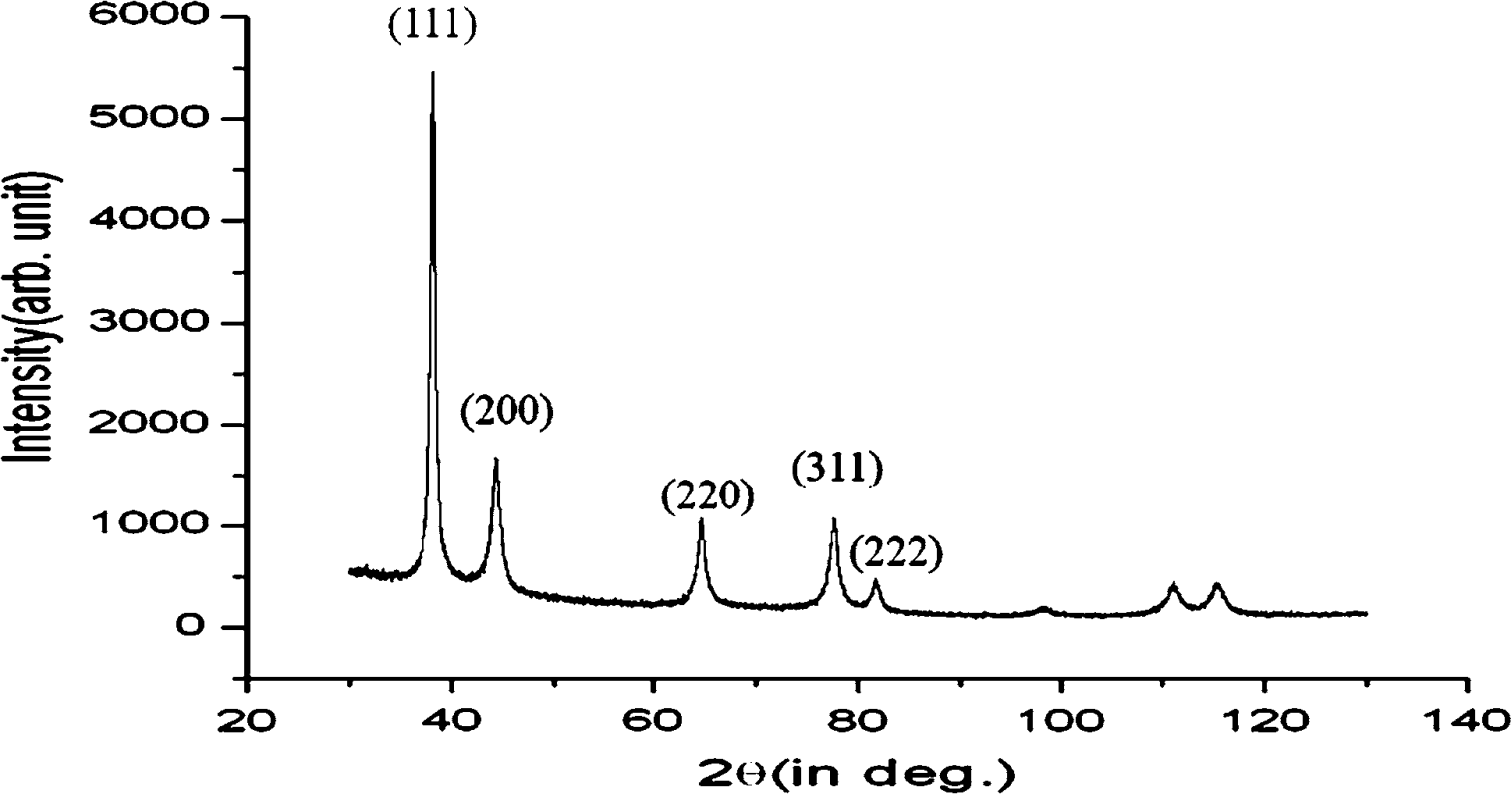
**Representative XRD patterns of gold nanotriangles synthesized by the reaction of 1 mM aqueous HAuCl_4_ solution with endophytic *A. clavatus* biomass**.

**Figure 7:**
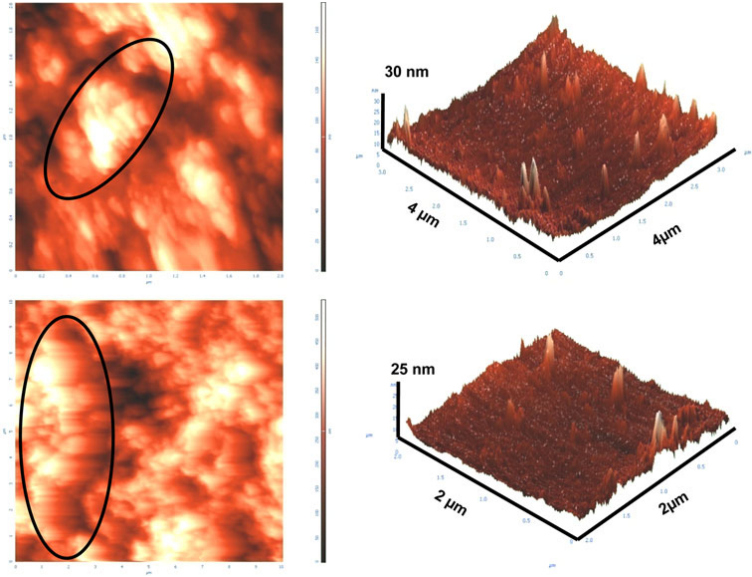
**AFM images of gold nanotriangles synthesized by the reduction of 1 mM HAuCl_4_ by endophytic *A. clavatus* after 48 h (a–b) and 72 h (c–d)**. Vertical stacking and clustering was observed with advancement of reaction period (**a**, **c**) within nanoparticles. Surface roughness of the nanoparticles was measured 25–30 nm (**b**, **d**).

**Figure 8:**
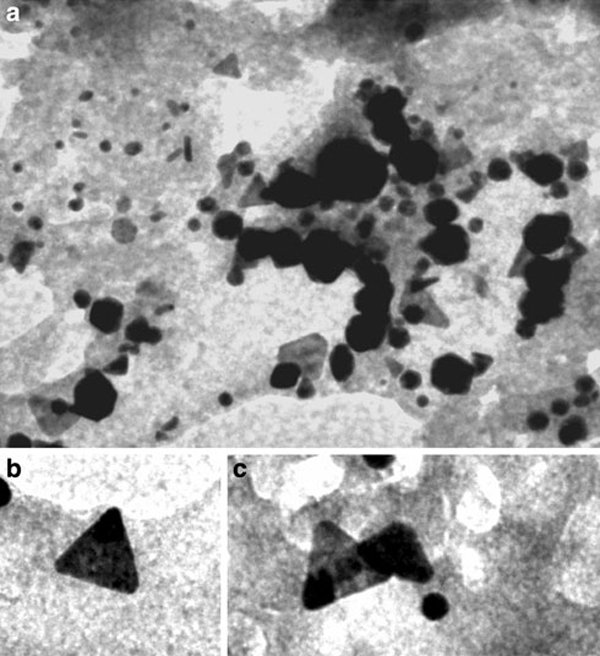
**Thin section of mycelium of *A. clavatus* after treatment with 1 mM HAuCl_4_ for 72 h. The polydispersity in morphology of the gold nanoparticle was observed (a–c)**. However, triangular nanoparticles are dominantly observed with variation in shape and edge margins (**a**–**b**) (*Scale bar* 200 nm for **a**, and 50 nm for **b**, **c**).

The endophytic *Aspergillus clavatus* strain was isolated from sterilized stem tissues of *Azadirachta indica*. This strain is identified using modern taxonomic keys with microscopic observations (Figure 1). One can observe the conidiophore with the naked eye, since this species of *Aspergillus* possesses largest conidiophores among the *Aspergillus* spp. (Figure 1a), the club-shaped conidiophores are 2–4 mm in length, stipes smooth-walled hyaline (inset Figure 1c). Conidial heads radiate, later splitting into several columns. Vesicles are clavate, 40–60 mm diameter conidiogenous cells are uniseriate, conidia smooth walled, pale green, ellipsoidal, 7–8 × 2–3 micrometre (Figure 1b, c). The morphotaxonomic keys of this fungus was so strong that one does not require any molecular identification. When the biomass of fungus was challenged with 1 mM HAuCl_4_ aqueous solution, a rapid change in the colour of the biomass was observed from fresh white to the dark purple (Figure 2), similarly the reaction mixture for fungal extract was also observed (inset, Figure 2). This change in colour was due to the collective coherent oscillation of conduction electrons at the surface of the gold nanoparticles when these particles interact with the oscillating electric field of the incident light, a phenomenon called surface plasmon resonance (SPR). This change in colour indicates that reduction in AuCl_4_^-^ ions takes place. When this reaction was traced with UV–vis spectroscopy, gold SPR bands were observed at *ca.* 540 nm, which steadily increases in intensity as a function of time of reaction. A 72-h reaction mixture has greater absorption intensity at *ca.* 540 than 48 h reaction mixture (Figure 3). The surface plasmon bands for the gold nanoparticles usually ranges between 510 and 560 nm in aqueous solution depending upon the function of their morphology, since plasmon bands are very sensitive to the length and sharpness of the tips of nanomaterials. The spherical nanoparticles, however, have strong absorption at about 520 nm with almost no absorption after 600 nm; however, the triangular shape has absorption at 540 which extends well in near infra red region (NIR). At maturation of reaction, the wavelength of surface plasmon bands stabilizes at 555 nm (Figure 3). Thus, the wavelength of peak absorption depends upon several factors such as particle size, dielectric constant of surrounding media and the inter-particle distance [19]. The representative bright field TEM images (Figure 4a, b) shows a relatively large population of flat gold nanotriangles along with some spherical and hexagonal gold nanoparticles formed by the spontaneous reduction in aqueous chloroaurate ions (AuCl^-4^) by fermentation extract of *A. clavatus*. The images also confirm that the gold nanotriangles are single crystalline in nature and could be indexed based on the face-centred cubic (fcc) structure of gold. The purified gold nanotriangles showed a particle size distribution ranging from 20–35 nm with an average particle size of 30 ± 2 nm (inset Figure 4b), this indicates that distribution is monodispersed. Consequently, we obtained a much higher population of nanotriangles in comparison with other morphologies, this encourage us to go deep into the optimization of parameters to get a control over the shape. All the nanotriangles observed under low-resolution TEM were ostensibly flat (Figure 4c–h), and showing tip truncation and rounding from sharp angle (Figure 4c, f, h) to sniped angle (Figure 4d, g) nanotriangles. When significant rounding (snipping) occurs, these nanostructures remains no longer as triangular nanoprisms and generally transformed into nanodisks or in cases of truncation without rounding, hexagonal nanoprisms. Synthesis of gold nanotriangles are kinetically driven process and is a result of aggregation and rearrangement of smaller size particles, which act as a nuclei for further growth into anisotropic triangular structures. It is also observed that the low rate of reduction in metal ions at normal room temperature possibly facilitate the growth of anisotropic nanoparticles, and with slight modifications in the temperature and the reaction medium one can be enabled in fabricating the well-defined triangular gold nanoparticles [20]. We have made efforts for the measurements of comparatively large, single gold nanotriangles (inset Figure 5), the height of the nanotriangles was about 140 nm, however, the facets length varies from 130 to 350 nm (Figure 5). But this may not be the case for all the nanotriangles, some has heights as low as 25–30 nm (Figure 7b, d). The AFM studies showed a maximum height of smallest nanotriangle was within 30 nm and the thickness ranges in between 2–8 nm (Figure 7b, d), and that all the triangles observed are equilateral with flat surfaces. In order to confirm the monocrystalline nature of the gold nanoparticles, XRD analysis was performed. Figure 6 shows the X-ray diffraction pattern obtained from the gold nanotriangles. The Bragg reflections obtained from the gold nanotriangle clearly correspond to the fcc crystalline structure of gold. The XRD pattern exhibits four identical diffraction peaks corresponding to the [111], [200], [220] and [311] appearing at 2θ = 38*.*2°, 44*.*5°, 65*.*6° and 78*.*6° of metal gold, respectively, (International Centre for Diffraction Data, ICDD No. 4–0783), indicating that the precipitate is composed of pure crystalline gold (Figure 6). As per the XRD pattern, a very intense Bragg reflection for the [111] lattice is observed, suggesting that the [111] oriented gold nanotriangles are lying flat [21] on the planar surface, while the reflections correspond to [220] and [311] with lattice spacing of 1.44 and 1.23 A° is specific for the triangular morphology, respectively. It is also notable that the ratio of intensity between the [200] and [111] diffraction peaks for the prepared sample is much lower than the standard (0.042 vs. 0.33), and this rationally decreases as the particles size increases. These observations confirm that as-formed gold nanoparticles are primarily dominated by [111] facets [22], which are quite consistent with the above electron diffraction observation. AFM has been performed on the glass substrate in contact mode, for the two reaction mixture one of 48 h and another was 72 h incubation periods. The Figure 7b, d shows heterogeneous vertical and lateral dimensions since stacking defaults of gold nanotriangles takes place due to longer incubation of the reaction mixture. The three-dimensional images of these nanostructures show surface roughness of about 30 nm (Figure 7b) and 25 nm (Figure 7d), interestingly stacking faults of nanotriangles was observed as they grow in size, which results in an overall increase in the surface roughness. The Figure 7a, c also tested and verified the results already obtained in short incubated samples. The gold nanoparticles are not resolved into single crystals due to the vertical stacking of nanotriangles, and thus the measurement and estimation of size for single nanoparticle with AFM remains intricating. Although the exact mechanism of the synthesis of nanomaterials are not known, but it was observed that when fungal biomass was treated with 1 mM aqueous solution of HAuCl_4,_ there are negligible amount of nanoparticles present in the solution (Figure 2), the biomass instead changed into purple colour, this clearly indicates that the reduction in gold ions takes place intracellular (surface reduction). For getting some more insight into the exact mechanism of bioreduction, a TEM analysis of fungal cells had been performed that are challenged with aqueous gold ions for 72 h. The thin sections of fungal cells shows the presence of plenty of triangular, hexagonal and spherical gold nanoparticles bound to the surface of the cells (Figure 8a–c); however, the triangular nanoparticles are relatively smaller in size than the spherical and other shapes. The presence of these shapes indicates that many particles are not transformed into triangle (Figure 8b) due to lack of nucleation or undergone surface reorganization in such a way that they no longer exhibit the ideal triangular nanostructures. A lot of research in green synthesis of nanomaterials are currently focused on the mechanistic approach to define the mechanism involved in this process [22-24]. A huge number of microbial strains are screened for their potential of producing metal nanoparticles; however, the mechanism of biosynthesis has not been established yet. The metabolic convolutions of viable microorganisms are even more complicating the process of analysis and identification of active species in the nucleation and growth of metal nanoparticles. Many researchers, however, speculated about the role of secretary enzymes such as NADH-dependent reductase of microbial origin, which may be responsible for the reduction in metal ions for growth and nucleation of nanoparticles [25,26]. However, the biochemical mechanism of metal ion reduction and the subsequent NP formation remain unexplored and need further research. Thus, thorough and in-depth understanding of the biochemical mechanisms associated with nanomaterials biosynthesis is needed. The analysis and identification of active constituents with HPLC and other analytical tools are required to solve the mystery of nucleation and growth of metal nanoparticles in the microbial system.

## Conclusions

In summary, we have demonstrated the shape controlled biosynthesis of gold nanotriangles using endophytic fungi *Aspergillus clavatus*, isolated from surface sterilized stem tissues of *Azadirachta indica* A. Juss. Results showed that triangular gold nanoparticles are formed along with some spherical as well as hexagonal morphology. It was also observed that the synthesis of gold nanotriangles are extracellular and showing a high aspect ratio. The study reported herein serve as a unique single-step green protocol for the generation and stabilization of nontoxic gold nanotriangles (GNT), exploitable in a myriad of diagnostic and therapeutic applications. *A. clavatus* induced synthesis of GNT will provide unprecedented opportunities towards the design and development of engineered 'green' gold nanotriangles that can be widely utilized in biomedical applications.
