# Correction: Biofabrication of anisotropic gold nanotriangles using extract of endophytic *Aspergillus clavatus *as a dual functional reductant and stabilizer

**DOI:** 10.1186/1556-276X-6-261

**Published:** 2011-03-28

**Authors:** Vijay C Verma, Ravindra N Kharwar, Santosh K Singh, Ravindra Solanki, Satya Prakash

**Affiliations:** 1 Centre of Experimental Medicine and Surgery, Institute of Medical Sciences, Banaras Hindu University, Varanasi-221005, India; 2 Mycopathology and Microbial Technology Laboratory, Centre of Advanced Study in Botany, Banaras Hindu University, Varanasi 221 005, India; 3 School of Material Science and Technology, Institute of Technology, Banaras Hindu University, Varanasi-221005, India; 4 National Facility for Tribal and Herbal Medicine, Institute of Medical Sciences, Banaras Hindu University, Varanasi-221005, India

## Correction

Ravindra N. Kharwar was inadvertently omitted from the author list of this article [1]. The correct author list is displayed above.

The Acknowledgement is also amended as follows:

This work is part of the PhD thesis of VCV, and was financially supported by the Council of Scientific and Industrial Research (*CSIR-09/013(205)/2008/EMR-I, dt.28-09-2008*), New Delhi India. Authors are thankful to the Professor-in-charge, Centre of Experimental Medicine and Surgery (CEMS), and Head Department of Botany, Banaras Hindu University. Authors also extend their thanks to Prof. Dhananjai Pandey, School of Material Science and Technology, Institute of Technology, Banaras Hindu University for assistance with the XRD and AFM studies and to Dr. Madhu Yashpal, Scientist-in-charge, Electron Microscopy Facility, Department of Anatomy, Institute of Medical Sciences, Banaras Hindu University, India, for the TEM analysis of the gold nanoparticles.
